# High Ethanol Contents of Spirit Drinks in Kibera Slums, Kenya: Implications for Public Health

**DOI:** 10.3390/foods6100089

**Published:** 2017-10-17

**Authors:** Alex O. Okaru, Kennedy O. Abuga, Isaac O. Kibwage, Dirk W. Lachenmeier

**Affiliations:** 1 Department of Pharmaceutical Chemistry, University of Nairobi, Nairobi P.O. Box 19676-00202, Kenya; koabuga@uonbi.ac.ke (K.O.A.); okibwage@uonbi.ac.ke (I.O.K.); 2 Chemisches und Veterinäruntersuchungsamt (CVUA) Karlsruhe, Weissenburger Straße 3, 76187 Karlsruhe, Germany; lachenmeier@web.de

**Keywords:** artisanal spirit, alcoholic strength, volatiles

## Abstract

Cheap licit and artisanal illicit spirit drinks have been associated with numerous outbreaks of alcohol poisoning especially with methanol. This study aimed to evaluate the quality of cheap spirit drinks in Kibera slums in Nairobi County, Kenya. The samples consisted of cheap licit spirits (*n* = 11) and the artisanal spirit drink, ‘*chang’aa’*, (*n* = 28). The parameters of alcoholic strength and volatile composition were used as indicators of quality and were determined using gas chromatography with flame ionization detection (GC-FID) and gas chromatography-mass spectrometry (GC-MS) respectively. The ranges for alcoholic strength were 42.8–85.8% vol and 28.3–56.7% vol for *chang’aa* and licit spirit drinks respectively, while the pH ranges were 3.3–4.2 and 4.4–4.8 for *chang’aa* and licit spirit drinks respectively. The majority of volatiles were found in artisanal spirits and they included higher alcohols, ethyl esters and carbonyl compounds. The alcoholic strength of all the artisanal spirits (100%) and 91% of the licit spirits was above the 40% vol of standard spirits such as vodka. The high ethanol content of the alcohol products was the only element of public health significance in this study.

## 1. Introduction

The World Health Organization (WHO) categorizes alcoholic beverages into recorded and unrecorded alcohol products [1]. Recorded alcohol products are those whose consumption is registered and licit while unrecorded alcohol includes homemade and artisanal drinks such as *chang’aa*, unregistered or counterfeited drinks and non-beverage or surrogate alcohols derived from medicinal products, automobile products or cosmetics [1,2,3]. *Chang’aa* is an artisanal illicit spirit drink obtained from distillation of liquor from fermented maize grains. The alcoholic content of *chang’aa* is enhanced by addition of sucrose to the fermenting mash before distillation. The production and consumption of the artisanal spirit was first banned in Kenya in 1980 through the *Chang’aa* Prohibition Act of Kenya but due to the failure to mitigate against the harm arising from the drink, it was legitimized in 2010 by the Alcoholic Drinks Control Bill of 2010 of Kenya. The bill sought to regulate the *chang’aa* industry with hopes of lowering the prevalence of alcohol poisoning. The Kenya Bureau of Standards (KEBS), a government standards body, also introduced regulation KS 2326:2011 [4] for the spirit.

According to the WHO estimates, the average adult (15+ years) per capita consumption of unrecorded alcohol is 2.5 L for Kenya (total per capita consumption is 4.3 L) corresponding to 58% [5]. Epidemiological evidence attributes 4% of the global burden of disease to alcohol and there exists a causal relationship of alcohol with more than 60 diseases such as malignant neoplasms, neuropsychiatric disorders, gastrointestinal diseases and diabetes mellitus, liver cirrhosis, injuries and psychosis [6,7] and the high content of ethanol has been cited as the main concern for public health in regard to unrecorded alcohol [3,8,9,10].

Ethanol and other congeners in spirit drinks can be measured by a variety of methods such as densimetry [11], Fourier transform infrared spectroscopy [12] nuclear magnetic resonance spectroscopy [13], refractometry [14], ultraviolet (UV)-visible (Vis) spectrophotometry [14], enzymatic methods [15], gas chromatography [14,16], high performance liquid chromatography [17], Raman spectroscopy [18,19,20] and flow injection analysis [15,21,22] among others with the choice of method depending on the type of alcohol product being analysed.

Kibera slum is located 5 km southwest of Nairobi Central Business District and is characterized by poor sewerage and drainage systems and lack of piped drinking water. The consumption of illicit brews and methanol-laced spirit drinks has been widely reported in Kenya [23] and socially deprived communities such as slum dwellers are more likely to consume such alcohol products. Indeed, the majority of consumers of alcoholic beverages in this low socio-economic setting are more likely to rely on cheap spirit drinks due to economic constraints and the ready availability [24].

Therefore it is important to evaluate the quality of the alcoholic beverages consumed in Kibera slums in context of consumer safety. This study, therefore, aims to determine the content of ethanol and to qualitatively identify the volatile congeners of cheap spirit drinks in the Kibera slums.

## 2. Materials and Methods

### 2.1. Samples

Twenty eight *chang’aa* samples were obtained from various villages within the Kibera slums. Aliquots measuring 100–200 mL were obtained from each site sampled. The samples were collected in the months of April and May 2015. For security reasons, a guide was used to locate the *chang’aa* selling households within the sprawling slums. The *chang’aa* samples were collected into clean plastic bottles and then coded for blind testing. Licit spirit drinks (*n* = 11) were obtained from Soweto and Laini Saba villages of Kibera slums.

### 2.2. Chemicals

High purity water was obtained by distillation using an Aquatron Automatic water still A4000 (Bibby Scientific, Staffordshire, UK) while analytical grade ethanol (99.9% *v*/*v*) and acetic acid (99.0% *v*/*v*) used as a working reference standards were from Scharlau (Sentmenat, Spain). Methanol (99.9% *v*/*v*) and *n*-amyl alcohol (99.9% *v*/*v*) were from Sigma-Aldrich (Steinheim, Germany) while acetaldehyde (99.8% *v*/*v*), ethyl acetate (98.0% *v*/*v*), *iso*butanol (99.0% *v*/*v*) and *iso*amyl alcohol (99.0% *v*/*v*) were from Merck (Darmstadt, Germany).

### 2.3. Alcoholic Strength

The ethanol content was determined using gas chromatography (GC) with flame-ionization detection (FID). A Shimadzu GC-2010 plus (Shimadzu Corporation, Tokyo, Japan) gas chromatograph operated using GC solution software version 2.42 (Shimadzu Corporation, Tokyo, Japan) with a ZB-WAX plus column (60 m × 0.25 mm i.d., film thickness 0.25 µm (Phenomenex, Torrance, CA, USA) and flame ionization detector was used. Temperature program used was as follows: 40 °C hold for 7.5 min, 4 °C/min to 200 °C, hold for 5 min, 15 °C/min to 220 °C hold for 5 min. The temperature of the injection port and detector were set at 260 °C. Nitrogen was used as the carrier gas at a flow rate of 2 mL/min. The method was validated with respect to linearity, limit of detection (LOD), limit of quantitation (LOQ), precision and recovery in accordance to ICH guidelines [25].

The sample preparation is as described in the EC regulation 2870/2000 for analysis of volatiles and *n*-amyl alcohol was used as internal standard. In brief, 9.0 mL of the beverage and 1.0 mL of *n*-amyl alcohol were pipetted into a 100 mL volumetric flask and made up to volume with deionised water. The resultant mixture was vortexed before injection into the gas chromatographic system. One microliter of each sample was injected into the GC-FID system with a split ratio of 100:1. Triplicate injections were made for each sample and a coefficient of variation (CV) of the peak areas determined. A CV < 3% value was considered adequate for the accurate determination of the alcoholic strength. Quantification was achieved by comparison of peak area ratios of the components to the internal standard against corresponding working reference standards. The range, mean, median and 95th percentile were used to describe the occurrence of the compounds in the alcoholic beverages.

### 2.4. Determination of pH

The pH of the alcohol spirits was determined on ‘as-it-is’ basis with a Jenway 3510 pH meter (Bibby Scientific, Staffordshire, UK).

### 2.5. Volatile Composition and Quantification

All samples were screened for volatiles including flavor compounds using a Shimadzu QP2010 GC-MS (Shimadzu Corporation, Tokyo, Japan) operated using a GC-MS solution version 2.71 (Shimadzu Corporation, Tokyo, Japan). A split/splitless injector was used anda ZB-WAX plus column (60 m × 0.25 mm i.d., film thickness 0.25 µm (Phenomenex, Torrance, CA, USA). Temperature was programmed thus: 60 °C hold for 1 min, 10 °C/min to 190 °C, hold for 5 min, 10 °C/min to 220 hold for 15 min. The temperature of the injection port and detector were set at 240 °C.

The instrument was operated in the electron impact ionization mode at 70 eV taking scans from 0 to 500 *m*/*z* in a 1 s cycle. One microliter of each sample was injected in the splitless mode. The mass spectrum obtained was compared against the NIST I and II mass spectral libraries (Standard Reference Data Program, National Institute of Standards and Technology, MD, USA) for identity. A similarity index ≥98% was considered sufficient for identification of analyte compounds.

Besides ethanol, the volatile compounds namely acetaldehyde, acetic acid, ethyl acetate, 1-propanol, *iso*butanol and *iso*pentanol were quantified using the GC-FID method and sample preparation procedures described for alcoholic strength (Section 2.3) but the working standards were the respective test compounds. Similarly, triplicate injections were made for each sample and a coefficient of variation (%RSD) of the peak areas less than 3% was considered adequate for the accurate determination of the alcoholic strength. Quantification was achieved by comparison of peak area ratios of the components to the internal standard against corresponding working reference standards. The statistical parameters of range, mean, median and 95th percentile were used to describe the occurrence of the compounds in the alcoholic beverages.

## 3. Results and Discussion

### 3.1. Sample Distribution

It was noted that there were few outlets that sold the licit spirit drinks compared to *chang’aa*. The retail price for about 200 mL of *chang’aa* ranged between USD 0.50–1.00 while most of the licit spirit drinks measuring about 250 mL retailed at USD 1.00. A narrow range of similar and cheap brands of the licit spirit drinks were stocked across the retail outlets visited.

### 3.2. Method Validation

The analytical performance of the GC method used for quantification gave acceptable validation parameters and was considered adequate for the determination of alcoholic strength and volatiles (Table 1).

### 3.3. Alcoholic Strength

The alcoholic strength of the *chang’aa* samples ranged from 42.8% vol to 85.8% vol with only eight samples (28.6%) out of 28 complying with the Kenya standard for *chang’aa* for alcoholic strength while the rest (71.4%) were above the acceptance criteria (35–57% vol). Similarly, 91% of the licit spirit drinks had a higher ethanol content than was labelled (range; 28.3% vol to 56.7% vol). The alcoholic strength of all the artisanal spirits (100%) and 91% of the licit spirits was above the 40% vol of standard spirits such as vodka. The drinks offered for sale within this slum are thus able to provide high amounts of ethanol in shorter dinking episodes and in smaller volumes thus being able to produce more pronounced intoxication effects. The high alcoholic strength in spirits poses public health risks to the consumers [2,3,7,10]. Therefore, there is need for consumer awareness on health hazards attributable to consumption of drinks with high ethanol content. Notably, high alcoholic strength up to 85.8% *v*/*v* in *chang’aa* samples peculiar since artisanal distillation being uncontrolled may not yield such high contents. Therefore it may be postulated that admixtures of pure ethanol occurred and that such *chang’aa* may be fraudulently be offered for sale as *chang’aa* in Kibera slums. However a comprehensive study using more samples would be required to prove this.

### 3.4. Analysis of pH

The pH range of the *chang’aa* and licit samples ranged between 3.3–4.2 and 4.4–8.8 respectively (Table 2 and Table 3). Among the licit drinks, brandies were slightly acidic while whiskey, gin and vodka were slightly basic. The low pH in brandies is associated with the presence of organic acids and sulfur dioxide or the use of mineral acids to adjust the pH [26] while slightly basic pH values may be attributed to treatment with alkalinizing agents to enhance the softness of the taste of the drinks [27]. The *chang’aa* samples were mildly acidic probably due the presence of organic acids such as acetic acid (Table 2) and succinic acid (Table 4) or acidic additives which were not determined during the study.

### 3.5. Volatiles Quantified 

Acetaldehyde may have arisen from inadequate hygiene and bacterial spoilage of the mashes and production equipment, use of yeast strains with a high production of acetaldehyde and oxidation of ethanol by O_2_ during the fermentation under aerobic conditions. Further oxidation of acetaldehyde may result in formation of acetic acid [28]. Acetaldehyde was detected in the *chang’aa* samples only in the range of 0.3–101 mg/100 mL of pure alcohol (p.a.) with a mean content of 17.5 mg/100 mL p.a. However, these levels were within the limits (126.4 mg/100 mL p.a.) set in the Kenyan standard for *chang’aa* (Table 2).

Determination of the methanol content is important because of the toxicity of its metabolites, formaldehyde and formic acid. Despite the numerous cases of methanol poisoning reported in Kenya, the current study did not detect methanol levels above the Kenyan limit of 5 mg/100 mL p.a. and European Union limit for vodka of 10 mg/100 mL p.a., respectively (mean content was found to be 1.4 mg/100 mL p.a.). The low levels of methanol are expected since the production process of the artisanal spirit involves natural fermentation of maize grains and use of high amounts of sugar. Methanol poisoning may only be caused by ad-mixture with the commercial solvent.

Ethyl acetate results from acetyl-CoA during fermentation because of the continuous oxidation of ethanol to acetic acid and the subsequent esterification [28]. Increased ethyl acetate and 1-propanol concentrations are indicative of prolonged storage of the raw material and probable acetic bacterial spoilage. The highest concentration of ethyl acetate in the *chang’aa* samples was 3.9 mg/100 mL p.a. with a mean of 0.7 mg/100 mL p.a. No ethyl acetate was detected in the licit brew samples. Nevertheless, all the *chang’aa* samples complied with the Kenyan limit for ethyl acetate. The mean content of 1-propanol in *chang’aa* was 7.2 mg/100 mL p.a. (range 1.0–37.7 mg/100 mL p.a.). The Kenyan limit for higher alcohols in *chang’aa* is qualitative since it prescribes that no precipitate shall be formed. However, artisanally and naturally produced spirits from grains and sugars and even commercially rectified spirits always contain some amount of higher alcohols. Therefore, all the *chang’aa* samples analyzed did not comply with Kenyan limit but are still judged as of no concern to public health [29].

*Iso*butanol (2-Methyl-1-propanol) concentration in the *chang’aa* was in the range of 0.5–15 mg/100 mL p.a. with a mean of 3.8 mg/100 mL p.a. while the *iso*amyl alcohol was in the range of 7.6–307 mg/100 mL p.a. with a mean of 67.3 mg/100 mL p.a. (Table 2). One sample of the licit drinks contained *iso*pentanol (1.7 mg/100 mL p.a.) (Table 3). *Iso*pentanol is formed during fermentation by deamination and decarboxylation reactions from isoleucine [30]. Elevated concentrations of *iso*amyl alcohol contribute negatively to the aroma of spirit drinks [31]. The mean content of higher alcohols was 78.3 mg/100 mL p.a., which is the sum total of 1-propanol, *iso*butanol and *iso*pentanol, in *chang’aa* samples. However, this has limited public health significance since these levels are by far lower than the preliminary guideline of 1000 g/hL p.a. for the sum of all higher alcohols thatis associated with acute and chronic effects such as liver cirrhosis [32]. The level (1000 g/hL) is higher than the concentrations usually found in both legal alcoholic beverages and surrogate alcohols [33].

### 3.6. Volatiles Detected

The volatile congeners qualitatively detected included esters and carbonyl compounds and these are known to confer distinct characteristics to the products. The volatile congeners originate from flavoring agents, raw materials and the subsequent processes such as mashing, fermentation, distillation and aging. The relative concentrations of these compounds vary with some contributing to the flavor and odor of the alcohol products. Nonetheless, the concentrations of these agents may have little relationship to the perceived olfactory characteristics of a product [34]. The majority of the volatiles were observed in artisanal spirits compared to licit spirits and there were differences in ‘typicities’ of the volatile profiles of artisanal spirits since the starting materials and art of brewing differ among producers from different communities in the slums. Ethyl acetate, 1-propanol, *iso*butanol, *iso*pentanol, ethyl lactate, 2,3-butanediol and acetic acid were present in all samples of the artisanal spirits.

Carbonyl compounds result from spontaneous or microbially-mediated oxidation. The carbonyls detected in the samples include acetaldehyde, acetone, acetoin, furfuryl alcohol, 5-hydroxymethyl furfural and furfural (Table 4). Furfural (2-furfural) and 5-hydroxymethyl furfural (HMF) are furanic derivatives formed during distillation due to dehydration of residual fermentable pentose sugars, xylose and rhamnose, respectively. The dehydration is caused by unfavorable fermentation conditions such as heating in acid conditions and/or Maillard reaction [35]. In our study, furfural was detected in ten *chang’aa* samples while 5-HMF occurred in only one of the licit spirit drinks, C13. This could be attributed to the uncontrolled distillation conditions employed in the production of *chang’aa*. Phenylethanol, a tail fraction, was detected in 26 of the 28 *chang’aa* samples while it was not detected in the licit spirit drinks (Table 4). This could be attributed to the inefficient distillation conditions employed in the production of *chang’aa.* Other components detected in licit spirit, C24, were benzyl alcohol and α-terpineol, a terpenoid used as a flavoring agent.

Esters are responsible for the sensory characteristics of spirits, giving them a pleasant fruity smell and they arise during fermentation processes of organic acids and alcohols. Ethyl esters of fatty acids are the most important aroma compounds in the spirit drinks. They are enzymatically produced during yeast fermentation and from ethanolysis of acyl-CoA that is formed during fatty acids synthesis or degradation [36]. Six ethyl esters were identified in illicit spirits and one in licit spirit drink, C16, namely ethyl caprylate, ethyl acetate, ethyl butyrate and ethyl caproate. Ethyl lactate serves to stabilize the distillate flavor and softens the harsh flavor characteristics present in low concentrations. The presence of lactic acid bacteria increases its concentration and contributes negatively to the distillate organoleptic quality [36]. Ethyl lactate was detected in all *chang’aa* samples and but not in licit spirit drinks.

### 3.7. Public Health Implications

The consumption and production of traditional drinks such as *chang’aa* is reported to be high in sub-Saharan Africa [37]. These products are not labelled about their alcoholic strength and thus consumers may use organoleptic characteristics to make a judgement on ethanol content, which is difficult if impossible, however [10]. Research has shown that that consumers tend to ingest more volume of alcohol [10] and therefore are at a greater risk for health due to high ethanol amounts.

Although high alcoholic strengths have been reported in other studies conducted in Poland [10], it is worth noting that artisanal distillation of grains to produce *chang’aa* would certainly not achieve such high alcoholic strengths that have been reported in this study. Plausibly, these extreme ethanol strengths may be from admixtures of rectified industrial ethanol into the traditional spirit, *chang’aa*. Further comprehensive study is required to investigate this finding using more samples.

## 4. Conclusions

This study in Kenya, which found extreme and unlabeled alcoholic strengths in unrecorded spirits, corroborates results from other countries (see [38,39]) namely that the only common element is the higher alcoholic strength of unrecorded products compared with licit spirits. The public health relevance of this observation is especially grave because the higher content of ethanol is not labelled on the products and thus the consumer may ingest more alcohol than with recorded spirits.

## Figures and Tables

**Table 1:** Method validation.

Parameter	Component							
	EtOH	Acetaldehyde	Acetic Acid	MeOH	Ethyl Acetate	1-Propanol	*Iso*butanol	*Iso*pentanol
LOD ^a^	0.02	0.03	0.02	0.02	0.02	0.03	0.02	0.03
LOQ ^a^	0.07	0.08	0.05	0.04	0.07	0.09	0.06	0.08
Precision ^b^	1.8	3.2	2.6	1.1	3.4	3.1	3.7	2.1
Recovery ^c^	100.2	100.5	100.4	102	98	101.3	99.5	98.7

a—mg/100 mL; p.a., b—% RSD; c—% mean.

**Table 2:** Analysis results of *chang’aa* samples.

Sample	Alcoholic Strength (% vol at 20 °C)	pH	mg/100 mL Pure Alcohol (p.a.)							
			Acetaldehyde	Acetic Acid	Methanol	Ethyl Acetate	1-Propanol	*Iso*butanol	*Iso*pentanol	Higher Alcohols
K01	66.1 ± 0.22	3.3	58.6	29.6	1.7	0.8	12.9	7.4	93.2	114
K02	85.8 ± 0.32	3.8	44.5	32.2	1.8	0.3	4.3	2.4	25.5	32
K03	76.0 ± 0.17	3.9	32.6	13.3	2.5	0.3	12.8	4.6	124.8	142
K04	58.7 ± 0.25	3.7	100.6	43.6	5.1	2.5	23.2	11.4	160.6	195
K05	63.6 ± 0.36	3.7	101.0	46.2	3.2	3.1	27.8	14.1	191.9	234
K06	60.5 ± 0.59	3.8	46.6	20.5	4.4	3.9	22.6	13.3	229.2	265
K07	72.7 ± 0.33	3.9	36.0	3.6	4.2	1.5	37.7	5.7	279.8	323
K08	48.8 ± 0.38	3.8	34.8	12.2	4.2	2.3	13.3	14.5	306.5	334
K09	68.8 ± 0.10	4.2	24.8	21.0	1.9	1.1	2.0	13.4	71.9	87
K10	49.7 ± 027	3.7	1.1	2.7	ND	0.3	2.2	1.0	17.8	21
K11	62.5 ± 0.20	3.8	0.3	0.1	ND	0.2	2.6	1.1	25.8	29
K12	45.5 ± 0.23	3.7	0.8	1.0	0.1	0.2	2.0	1.0	15.5	18
K13	59.7 ± 0.11	3.9	0.5	1.2	0.3	0.2	1.9	2.7	63.9	69
K14	70.7 ± 0.62	3.8	1.5	1.1	ND	ND	1.6	0.9	8.2	11
K15	58.0 ± 0.69	3.6	3.2	2.7	0.2	0.2	1.3	0.9	15.8	18
K16	53.3 ± 0.48	3.6	3.6	2.4	0.4	0.2	1.7	0.7	13.2	16
K17	76.9 ± 0.92	4.1	2.1	0.3	0.4	0.2	2.0	0.9	14.1	17
K18	59.1 ± 0.63	3.9	1.7	0.9	0.1	0.2	1.2	1.2	26.9	29
K19	42.8 ± 0.56	3.6	2.0	0.7	0.1	ND	1.0	0.7	15.8	18
K20	59.0 ± 0.34	3.9	1.7	2.2	0.5	0.2	2.3	0.9	14.9	18
K21	68.1 ± 0.11	4.2	0.6	0.3	0.8	ND	5.1	0.9	16.9	23
K22	76.2 ± 0.63	4.2	0.6	0.1	0.9	ND	6.5	1.1	23.9	31
K23	63.6 ± 0.51	3.9	0.9	0.7	0.5	0.1	2.2	2.2	59.9	64
K24	49.7 ± 0.55	3.9	0.8	0.2	0.5	ND	3.6	0.5	10.5	15
K25	54.6 ± 0.85	4.1	0.5	0.4	0.3	ND	2.0	0.5	7.6	10
K26	74.4 ± 0.11	3.9	0.7	0.4	0.5	0.9	2.0	0.8	15.3	18
K27	66.6 ± 0.40	3.8	0.4	0.6	0.5	0.2	2.1	0.9	26.1	29
K28	51.6 ± 0.39	3.3	2.4	2.8	0.1	0.3	1.2	0.5	9.2	11
Range	42.8–85.8	3.3–4.2	0.3–101	0.1–46	ND–5.1	ND–3.9	1.0–37.7	0.5–14.5	7.6–307	10–334
Median	61.5	3.8	1.9	1.7	0.5	0.3	2.2	1.0	24.7	29.3
Mean	62.3 ± 10.64	3.8	18.0	8.7	1.4	0.9	7.2	3.8	67.3	78.3
P95	81.8	4.2	85.9	39.6	4. 3	3.1	26.2	13.8	262.1	302.9
Kenyan limit	35–57	-	126.4	-	5	580 *	-	-	-	-

ND—below LOQ, Higher alcohols were calculated by the sum of 1-propanol, *iso*butanol (2-methyl-1-propanol) and *iso*amyl alcohols, P95 is the 95th percentile of values, - limits not established for the parameter, * total esters expressed as ethyl acetate.

**Table 3:** Selected analytical results of licit spirits.

Sample	Type	Alcoholic Strength (% vol at 20 °C)	pH	mg/100 mL Pure Alcohol	
				Methanol	*Iso*pentanol
C02	Gin	41.7 ± 0.32	7.6	1.0	ND
C03	Vodka	28.3 ± 0.19	7.8	0.4	ND
C07	Brandy	45.8 ± 0.33	4.8	ND	ND
C08	Brandy	49.1 ± 0.38	4.8	0.1	ND
C13	Vodka	56.7 ± 0.15	7.7	ND	ND
C14	Vodka	49.7 ± 0.49	8.5	ND	ND
C16	Brandy	56.1 ± 0.42	4.4	0.1	1.7
C17	Vodka	50.4 ± 0.90	8.1	0.04	ND
C23	Vodka	57.2 ± 0.52	8.8	0.6	ND
C24	Gin	51.3 ± 0.47	8.4	0.4	ND
C25	Vodka	48.7 ± 0.34	7.7	ND	ND

ND—Below LOQ.

**Table 4:** Volatile constituents identified in *chang’aa* samples.

Sample	Sample Composition										
	ACA	ACO	ACT	2,3-Bu	ACON	ECPL	ECPN	FA	Suc	EL	EPT
RT (min)	5.3	5.8	6.2	7.0	11.7	13.7	16.9	17.2	17.4	20.3	28.8
K01	-	-	-	-	-	-	-	-	-	-	-
K02	+	-	-	-	-	-	-	-	-	-	-
K03	-	-	-	-	-	-	-	-	-	-	-
K04	-	-	-	-	-	+	+	-	+	-	-
K05	-	-	-	-	-	-	+	-	-	-	-
K06	-	-	-	-	-	-	+	-	-	-	-
K07	+	-	-	-	+	-	+	+	-	+	+
K08	+	-	-	-	-	-	-	-	+	-	-
K09	-	-	-	-	-	-	-	+	-	-	-
K10	-	-	-	-	-	-	+	+	-	-	-
K11	+	-	-	-	-	-	+	-	-	+	-
K12	+	-	-	-	+	-	-	-	-	-	-
K13	+	-	-	-	-	-	-	-	-	-	-
K14	-	-	-	-	-	-	+	+	+	+	-
K15	-	-	-	-	-	-	+	-	+	-	-
K16	-	-	-	-	-	-	+	-	+	-	-
K17	+	-	-	-	-	-	+	-	-	+	-
K18	+	-	-	-	-	-	+	-	+	+	-
K19	+	-	-	-	-	-	-	+	-	-	-
K20	-	-	-	-	-	-	+	+	-	+	-
K21	+	+	+	+	-	-	+	+	-	+	+
K22	+	+	+	+	+	+	+	-	-	+	+
K23	+	+	+	+	+	-	+	+	-	-	-
K24	+	+	+	+	+	-	+	+	-	-	-
K25	+	+	+	+	+	-	+	+	-	+	-
K26	+	-	-	+	-	-	+	-	-	-	-
K27	+	-	-	+	-	-	+	-	-	-	-
K28	+	-	-	+	-	+	-	+	-	-	-

ACA—Acetaldehyde, ACO—Acetone, ACT—Acetal, 2,3-but—2,3-butadione, ACON—Acetoin, FA—2-Furfural, ECPL—Ethyl caprylate, ECPO—Ethyl caproate, ECPN—Ethyl caprinate, EL—Ethyl laurate, EPT—Ethyl palmitate, EPD—Ethyl pentadecanoate, Hep—Heptanoic acid, Succ—Succinic acid and PhEt—Phenethyl alcohol, +—Detected, -—Not detected, Hep. was only detected in K28 while EPD was in K03 and K04, ECPO in K21.
